# Abrasion of the Teeth

**Published:** 1895-11

**Authors:** Carrie M. Stewart


### Abrasion of the teeth.

BY DR. CARRIE M. STEWART, D.D.S.

The simplest form of wasting away of the teeth is known as chemical or spontaneous abrasion.

Of this diseased condition little is known, excepting that it is likely to attact the teeth suddenly and unexpectedly, and apparently without cause. It may cease its destructive action after a time as suddenly as it began, or continue until the teeth affected are almost entirely destroyed, causing great pain and discomfort to the patient. Again, its progress may be very rapid or so slow as to require a number of years in which to complete it destructive work.

When affected by this condition, the teeth are found to be unusually sensitive to the action of acids, heat and cold and any other form of irritation to which they may be subject There is but little pain experienced, excepting when irritated, until the disease has reached the vicinity of the pulp, when this organ gives prompt notice of its danger of exposure from destruction of the enamel and dentine forming its normal protection. Of the cause of this condition, but little is known. It is not due to mechanical means for mechanical abrasion forms a class by itself and its cause and the remidy for its cure are easily understood and acted upon as the circumstances may require.

It was thought by some authors, to be due to absorption, but that theory cannot hold good from the fact that, if due to absorption, it would be extremely unlikely and highly improbable, that the pulp would make the calcareous deposition which it usually does, to protect itself from the action of external influences.

All agree in attributing the cause of the mischief to chemical action of some kind; but just what it is and in what part of the mouth it is to be found, has not yet been determined. Some have confined it to a peculiar acid condition of the saliva, but it is plainly to be seen that if this was the case all the teeth would be acted upon alike, which they are not. Just now, it is attributed to an acid condition of the mucous of the mouth, which, according to the statements of the advocates of this theory, remains in contact with certain parts of the teeth attacted and chemical abrasion of that part results.

This seems to me as improbable as the acid saliva theory. If it were possible for the mouth to be kept in a certain position all the time so that the acid mucous could act only on certain portions of the teeth, it might be quite probable that the result was due to such a cause, but such is not the case. Of course the teeth nearest the point where the secretion and exudation of the vitiated mucous takes place would be more or less affected; but the mucous does not remain at one point, it is distributed to other parts of the mouth as well as the saliva and comes continually in contact with portions of the teeth which are not affected by chemical abrasion. So it seems to me that the objections to the acid saliva theory would hold good in the acid mucous theory also. The superior teeth are more often attacted by this condition than the inferior. It usually begins on the points of the central incisors, progressing most rapidly in the median line and from there proceeding each way into the tooth substance. On account of the peculiar course it takes, the central incisors are sometimes found with their cutting edges cupped out leaving an elliptical space when closed. This form of the abrasion is of rare occurance however, and is supposed, by the upholders of the acid mucous theory, to be due to the action of the acid mucous secreted by the mucous follicles of the tongue, which organ, being brought frequently in contact with the cutting edges of these teeth, produces this peculiar form of the abrasion.

Chemical abrasion may be found on the labial surfaces of the superior incisors and cuspids, and the bicuspids and first permanent molar. The second and third molars are rarely affected. The buccal surface of the teeth are seldom acted upon.

This condition may be manifested on the masticating surfaces of the molars, especially upon their prominances. It may also be found in the shape of pits or grooves on various parts of the teeth.

One curious feature of this diseased condition is, that the surfaces affected are always smooth and highly polished, not presenting the roughened appearance that the action of acid upon the tooth substance usually does.

As we know nothing of the subject, excepting that it is a gradual wasting away of the enamel and dentine, varying in rapidity, cause unknown, its treatment is very difficult. Supposing it to be due to a vitiated condition of the oral secretions, the proper method of procedure would be to employ systemic treatment with a view to neutralizing or entirely abolishing this acid condition. Constitutional treatment is always more or less long and tedious however, and we do not know certainly that this condition absolutely requires it, but it should be used until either a cure or a failure, after persistant and patient effort, takes place. Meantime, the abraded surfaces should be filled with gold, preferably, and the contour of the natural tooth retained as well as is in the power of the operator to do it.

He who discovers the cause, prevention and cure of this disease will confer a lasting benefit upon humanity, for although it may not be termed of frequent occurance, still when it does take place its ravages are anything but desirable or to be looked upon as of little importance to one who appreciates the value of a sound, perfect set of teeth.

